# Examining Contemporary Human Dignity: How Human Rights Evolve and Reshape Health Justice

**DOI:** 10.7759/cureus.82316

**Published:** 2025-04-15

**Authors:** Bilal Irfan, Batool Jaber, Maisa Awwad, Tareq AlSourani, Abdallah Abu Shammala

**Affiliations:** 1 Michigan Alzheimer's Disease Center, University of Michigan, Ann Arbor, USA; 2 Center for Bioethics, Harvard Medical School, Boston, USA; 3 Department of Global Health Research, Horizons Academy, Ramallah, PSE; 4 Department of Education, Birzeit University, Birzeit, PSE; 5 Department of Education, King's Academy, Amman, JOR; 6 Department of Internal Medicine, European Gaza Hospital, Gaza, PSE

**Keywords:** global health governance, health equity, human rights frameworks, localizing health, transformative constitutionalism

## Abstract

Human rights did not emerge in a political or cultural vacuum; their origins in 20th-century legal frameworks and postcolonial power asymmetries continue to shape evolving notions of dignity and justice. Central to this evolution is the tension between civil and political rights, understood as immediately enforceable, and economic, social, and cultural rights, which are too often neglected or treated as merely aspirational. Securing health as a right requires both negative and positive state obligations, challenging simplistic divisions between public and private spheres and exposing how gendered, racialized, and class-based violence often remains hidden within private realms. Colonial legacies, neoliberal market forces, and structural inequalities further reinforce injustice by rendering marginalized groups undeserving of care. Grassroots movements, from AIDS activists to Indigenous women’s campaigns, demonstrate the capacity of collective action to transform law, policy, and social norms. A rights-based approach to health financing demands that societies treat healthcare as a public good and moral commitment rather than as a commodity. At the global level, target-driven frameworks like the Millennium Development Goals have achieved discrete gains but often fail to address the systemic drivers of inequity. Ongoing armed conflicts reveal how militarized violence decimates health infrastructure and entrenches disparities for generations. True health equity and democracy, therefore, require robust institutions, transparent governance, and inclusive participation, all underpinned by the recognition that rights are shaped and reshaped by lived struggles. Only through this holistic lens, one that integrates civil, political, economic, social, and cultural dimensions, can efforts to realize the promise of universal human dignity be brought into practice.

## Editorial

The link between the origin stories of human rights and their present-day legitimacy requires further examination. Contemporary social movements are somewhat shaped by the character of a particular state, especially in light of a state’s origin story in a post-colonial order. It is compelling to note that even the oft-championed Universal Declaration of Human Rights reflects the time period it was constructed in and did not emerge in a political or cultural vacuum. Instead, its legitimacy and several other international institutions rest on a hard-won overlapping consensus among diverse intellectual traditions, whether legal, religious, or philosophical. We see examples of complementary or rival interpretations of universal human rights encapsulated in documents such as the Cairo Declaration of Human Rights ratified by member states of the Organization of Islamic Cooperation, reflecting society-based ethical-moral paradigms. This origin story remains crucial, albeit imperfect and shaped by power asymmetries after World War II. It reminds us that human rights are not static natural law but evolving legal and moral commitments that draw their force from ongoing international dialogues [1]. These foundations, in turn, matter deeply for how societies conceive of democratic norms [2]. Then, democracy is more than just the presence of elections, as it somewhat relies on a system in which citizens can contest unjust policies, demand accountability, and secure fair distribution of resources. Without strong democratic institutions capable of incorporating economic and social rights, like health, alongside civil and political ones, formal freedoms risk being hollow [3].

That tension is especially visible in the distinction between civil and political (CP) rights and economic, social, and cultural (ESC) rights. Historically, CP rights have been given legal immediacy, as seen in the right to a fair trial, freedom from torture, and freedom of expression being understood as immediately enforceable, often requiring that the state not interfere. Meanwhile, ESC rights such as health, education, and housing have been framed in international law as obligations achieved progressively and subject to available resources. While this language recognizes the realities of finite budgets, it can also be exploited to deprioritize essential social guarantees [1]. What can be taken away is that both CP and ESC rights need a blend of negative obligations, such as freedom from interference, and positive ones, such as access and funding. For instance, the right to a fair trial costs money, so one must fund impartial courts and ensure legal representation, yet we rarely dismiss that right for budgetary reasons. Thus, it can be said that the historical tendency to sideline ESC rights as vague aspirations is at least partially misguided and often politically motivated.

The public-private divide likewise looms large. Where CP rights traditionally constrained the state in the public realm, ESC rights often involve family or personal spheres historically deemed private [1]. The implications of this are massive, and while torture in infamous prisons in Argentina or Syria was a public wrong as an example, violence that women and children suffer in domestic spaces has still been somewhat overlooked by mainstream international law. This divide in our attention obscures how systematic subordination in families, through child marriages, denial of reproductive autonomy, or domestic violence, can be just as destructive as state-sponsored abuse. In recent years, women’s and children’s rights campaigns tore down many of these conceptual walls, showing that private actors can violate human dignity and that states protect those within private spheres.

Turning to health, we see how profoundly our conception of healthcare, whether viewed as charity, market commodity, or a right, reflects broader societal values [2]. In the United States, the neoliberal shift of the 1980s replaced a fledgling sense of shared social responsibility with privatized insurance markets [1]. The result was entire neighborhoods, frequently segregated by race and class, where health outcomes plummet compared to more affluent zip codes just blocks away. It is evident that stigma and discrimination both aggravate these gaps and deter potential remedies. The stark gaps, depending on jurisdiction, socioeconomic status, and legal status, are evident even in areas such as Palestine, where each little enclave may have starkly different health outcomes than those in another enclave a few miles over. Areas served by more United Nations facilities or charity hospitals may be more connected to health infrastructure, with access at times being driven by socioeconomic status, which may even drive Palestinians in frequently targeted refugee camps into further ghettoization. The COVID-19 pandemic’s manifestation in many countries classified as developing countries is also food for thought. Rather than investing in robust public health systems, many governments during the recent pandemic often took a punitive approach, further marginalizing those who most needed care [4].

Ethnicity, social class, and gender remain principal, interconnected drivers of health disparities [2]. In the United States, the crackdown on substance abuse disproportionately targeted portions of communities racialized as Black or Brown communities, fueling mass incarceration rather than tackling root causes such as poverty, inadequate housing, or limited access to quality healthcare [1]. Perhaps what is clear is that this is no accident but rather stems from a worldview where some communities are labeled as undeserving, their suffering explained away as misfortune rather than injustice. Nevertheless, there is encouragement to be found in examples of AIDS activists who fought government inaction, that there is space to cultivate change, and highlight how structural racism, sexism, and classism can, in fact, shape health outcomes. These successes teach us that human rights, in both CP and ESC dimensions, must be invoked to dismantle policies that systematically dehumanize entire groups or deprive them of their right to healthcare. We cannot hope to understand human rights and adequately apply them if left to being purely lofty abstractions one aspires to reach. They are born of history, shaped by power, and continuously redefined by the struggles of real people, physicians fighting for the right to treat patients in Palestine without criminalization, communities combating infectious disease outbreaks and stigma, or families demanding water and housing. In reclaiming the promise of these rights, we can affirm that health justice is more than empathy or pity, for it demands concrete structures, laws, and democratic processes that ensure all can flourish.

Cultural, economic, and gender-based injustices have also found their way into the medical field, with discriminatory attitudes being reported toward certain minoritized or underprivileged groups in society. This can sometimes prevent them from obtaining the standard of care they otherwise would have. Additionally, with the rise of neoliberalism, healthcare became enveloped in the market, becoming a commercialized commodity rather than a right or an automatic service people should not have to pay for.

The end of the Cold War ushered in a significant reconfiguration of human rights norms across the globe, particularly in elevating ESC rights to a status on par with CP rights. The 1993 World Conference on Human Rights in Vienna was especially pivotal as 171 governments reaffirmed that all human rights are universal, indivisible, and interdependent. Freed from Cold War ideological gridlock, the United Nations promoted an integrated vision of human rights through trans-sectoral development conferences in Rio, Vienna, Cairo, and Beijing in the early 1990s. These gatherings were transformative because they addressed interrelated challenges, including environmental sustainability, gender equality, and social development. They served to solidify the notion that issues like health, gender-based violence, and education require structural, cross-cutting solutions [1]. They also enlarged civil society’s role as unprecedented participation rates by non-governmental organizations shaped the outcomes sought by reframing once-marginalized concerns, such as violence against women in private spheres, as core human rights violations.

Building on the recognition of environmental sustainability as a human rights concern, the exacerbating nature of the climate crisis showcases how deeply the realization of health rights is intertwined with the preservation of the environment. Climate change is more than an environmental catastrophe, as it challenges human rights by threatening fundamental rights to life, health, food, and shelter, especially among populations that have contributed least to global emissions. The concept of climate justice can thus be utilized to frame climate change to move into the domain of human rights, which demands accountability for historical and contemporary emitters and protective measures for the most affected communities. International institutions are beginning to reflect this perspective. In 2022, the United Nations formally recognized a universal right to a clean, healthy, and sustainable environment, affirming that preserving the environment is foundational to human dignity and health. Adopting this planetary health lens can aid in increasing the scope of health justice to encompass a global pluralistic society, while insisting that safeguarding human dignity in the 21st century requires urgent action to address degradation of the environment and its unequal toll on populations and human well-being.

An important feature of this era was the emergence of transformative constitutionalism in many Latin American countries, differing significantly from the constitutional model present in the United States. Meanwhile, the United States emphasizes negative liberties, such as rights to be free from state interference. New or reformed constitutions in Argentina, Colombia, and South Africa have embraced positive obligations as integral to a democratic state of law. These constitutions recognized ESC rights, including health, demanding that state institutions actively secure minimum social guarantees for citizens. Courts were reimagined not as passive arbiters but as engines for robust social change. For example, Colombia’s Constitutional Court’s treatment of health as an integral component for a dignified life eroded the arbitrary distinctions drawn between CP and ESC rights in practice [1]. This was seen through landmark rulings such as T-760/08, which mandated systemic healthcare reform to enforce universal accessibility, and T-859/03, which explicitly affirmed healthcare as an immediately enforceable right, irrespective of resource limitations. The United States, by contrast, lacks an explicit constitutional guarantee to health, and American courts rarely engage in resource-allocation questions.

Nevertheless, establishing a universal core content of the right to health has proved difficult. As a United Nations committee focusing on ESC rights articulated within some of their comments and subsequent statements, countries must ensure at least a minimum set of entitlements, such as providing essential food, basic shelter, and primary healthcare, even if there is a situation of resource scarcity [1]. All the same, though, each country’s social, economic, and epidemiological contexts shape what primary care or essential medicines look like in practice. Thus, the aforementioned committee uses dialogue with states to contextualize obligations, reversing the burden of proof so that governments must explain any significant shortfalls. This approach, while pragmatic, further displays the complexity of enumerating a global standard for even basic services. It is also unclear at the outset what level of resource scarcity obliges government responsibility one way or the other, as in how much scarcity would absolve a government of shouldering the responsibility of servicing basic needs. There are key geopolitical realities that may shape the actualized health outcomes and pursuits of populations, at times out of the ability of one government itself to change.

For example, dismantling healthcare infrastructure during armed conflict can result in long-term repercussions beyond the crisis. This is particularly evident in Syria, where medical and healthcare buildings were often subject to attacks during the civil war, leaving them inoperable. This left many Syrians without access to basic health services, aggravating existing vulnerabilities. Healthcare scarcity in war zones can severely impact aid toward civilians with traumatic injuries and also contribute to the spread of diseases, an increase in infant and maternal mortality rates, and the collapse of routine immunization programs. While international relief efforts may strive to address this gap, insufficient and inconsistent funding due to ongoing violence remains an obstacle in many parts of the world. As a result, even after a conflict concludes, these health disparities can linger for decades, exacerbating social inequality and impeding recovery efforts.

The forced sterilizations in Peru in the 1990s, as well as the obstetric violence, go to illustrate how health systems embed certain colonialist and discriminatory norms. Extreme structural adjustment policies and labor precariousness converged with deep-seated racism toward indigenous women to result in punishing quotas for permanent contraception that prompted gross violations of bodily autonomy. In some cases, denial of lifesaving obstetric care was compounded by paternalism, language barriers, and a lack of cultural respect. Health services designed within neoliberal frameworks further eroded accountability, as local providers, often under flexible contracts, were coerced by top-down targets. By the 2000s, the Peruvian state’s safe motherhood strategies again weaponized facility-based deliveries against women who distrusted the very system that had systematically abused them [1]. Similarly, in Palestine, obstetric violence is intimately related to the tools of a colonial attack against an indigenous population. The targeting of women of reproductive age specifically to prevent births, thereby reducing a native population in sheer numbers, is one such example of a gross violation of the right to health and, relatively, the right to life itself [5].

One lever for democratizing health systems, then, in areas not affected by genocide, is community-driven oversight and engagement. That is not to say that such community efforts have no place in war-torn regions or those impacted by ethnic cleansing, but their impacts may be varied under the extreme constraints of constant targeting. Indeed, even in Gaza, there are services in the partially operational hospitals and health clinics aimed at trying to map the attacks on pregnant women and providing counseling, offered by those suffering genocide themselves, and support, when possible. However, we can turn to the case of Peru, wherein governmental agencies tasked with overseeing civil and human rights protection collaborated with indigenous women’s groups to monitor reproductive health services. Such accompaniment strategies, which empower local leaders to observe and critique clinics, can dismantle some punitive policies. More broadly, however, transformative constitutional provisions like streamlined protection writs in Colombia enabled some citizens to lodge claims for health entitlements directly before courts. Equally important, though, is bridging alliances among grassroots groups, civil rights movements, and mainstream human rights organizations, as it can yield formidable pressure on government policies, as seen in the advocacy that exposed Peru’s sterilization abuses. These coalitions must be based on mutual premises of human dignity, not sacrificial and driven by state or national politics over our shared humanity and quest for upholding rights.

Human rights and other civil rights communities have not always been aligned. In Latin America, historically strong ties between major human rights groups and certain religious groups often sidelined certain aspects of maternal health. Differences in access to specific medical procedures, in particular, have triggered and exacerbated existing tensions and divides [1]. Something similar holds in the United States, where mainstream civil liberties organizations at times focus on freedom of expression or due process but give less emphasis to structural gender or racial inequalities. Movement fragmentation can stem from ideological divides between religious or specific legal frameworks, making cross-coalition work fraught. At times, there is a need to gather together for a particular cause while recognizing pluralism in views and ideas. It can sometimes be meritorious to acknowledge that not all differences in views can be bridged, and not all coalitions need to be equated to reciprocal alliances. Groups hailing themselves as being from certain religious or ideological persuasions need not agree on their differences on specific social issues to be able to collectively champion against human rights abuses.

With all such advocacy work toward health progression and rights, there is a need to track meaningful and substantive progress. Yet we must be cautious about not relegating this one metric alone, as many factors may be used to assess change [1]. Legislative or judicial reforms, such as new protocols for informed consent, may only represent a fraction of change. Shifts in discourse, dismantling of racist stereotypes among health workers, and the empowerment of marginalized groups can be equally transformative, albeit harder to quantify. In a context where economic liberalization narrows political choice and deepens inequities, health rights advocacy must engage with formal law and the structural arrangements, labor conditions, funding priorities, or cultural biases that govern health systems. Only then can we meaningfully counter oppressive hierarchies and ensure that no one’s health decline or preventable loss of life is casually written off as a misfortune rather than an injustice demanding remedy.

The 2000s marked a pivotal shift in global development and health governance, in large part because of the HIV/AIDS pandemic’s devastating impact, particularly in sub-Saharan Africa, and the advent of the Millennium Development Goals (MDGs). Compared with the more expansive United Nations development conferences of the 1990s, which championed broad institutional reforms and social transformation, such as the Beijing Platform for Action, the MDGs introduced a more technocratic and target-driven approach. This narrower framework emphasized specific, measurable interventions, such as antiretroviral therapy for HIV/AIDS, without challenging the deeper structural factors, including poverty, gender inequality, and weak health systems, that in many ways underlie global health disparities [1].

The case of Malawi is particularly relevant in that we see how colonial legacies and global economic policies intersect to produce enduring forms of dependency. Even after achieving independence, Malawi was subjected to debt restructuring, austerity measures, and privatization mandates. These impositions severely limited the state’s capacity to build resilient health systems. Stories of women dying of childbirth complications in an under-resourced clinic that lacked even the most basic of emergency capacities illustrate how globalization under trial manifests at a personal, human level. The international intellectual property (IP) regime further compounds the issue as restrictive patent rules, set primarily by and for wealthier nations, dramatically inflate the cost of essential medicines, creating dependency and vulnerability in countries already reeling under the strain of debt owed to foreign investors [1].

A noticeable shift in governance for global health took shape during this period. HIV/AIDS became the catalyst for massive funding from new institutional actors, such as the Global Fund in 2002 and the United States’ President’s Emergency Plan for AIDS Relief in 2003. While these programs undeniably saved millions of lives, they also reinforced governance at a distance, wherein donor-driven priorities overshadowed local capacity-building. Furthermore, the revised International Health Regulations (IHR) in 2005 epitomized the global health security paradigm as it aimed to prevent the cross-border spread of disease while avoiding unnecessary interference with international traffic and trade. Although important in managing outbreaks like SARS-CoV-1 and later COVID-19, the IHR spotlighted a tension that protecting national borders from emerging threats can overshadow more endemic challenges, like malaria, tuberculosis, and maternal mortality, particularly in low-income countries [1].

Amid this shift in global health governance, the Treatment Action Campaign (TAC) in South Africa displayed how grassroots advocacy can reshape national and global structures. TAC acted to mobilize communities, litigated for expanded antiretroviral access, and pressured pharmaceutical companies to reduce prices and allow generic imports. Their court victories in the early 2000s, especially around preventing mother-to-child transmission of HIV, were landmark decisions that enabled defining how constitutional rights to health could become enforceable government obligations. Not only did TAC’s work facilitate broader access to HIV treatment, it also catalyzed international legal changes, most notably the declaration regarding public health that emerged from Doha in 2001. Although the declaration included language that gained some criticism, it nevertheless acknowledged that IP protections must yield to public health imperatives. This idea resurfaced during the COVID-19 vaccine and treatment debates [1].

Another theme worth dwelling on in more detail is the marked impact of colonialism and the concepts of culture and tradition in conceptualizing rights related to self-agency. International human rights institutions, especially after 2000, proliferated norms protecting such rights in detail, yet this expansion triggered backlash in some countries, where politicians weaponized specific conceptualizations to resist perceived Western impositions and reassert local culture [1]. These dynamics underline how health and human rights endeavors, whether related to HIV or broader health-related rights, often run up against powerful cultural narratives and vested interests that resist global rights frameworks. It also somewhat begs the question of whether there is a universal standard to human rights, and if so, who gets to decide and be a party to that framework. It also asks who has no seat or voice at the table.

There are a couple of lessons that can be drawn from here. One is that technocratic targets can yield rapid gains in narrow areas, such as HIV treatment, but may fail to address root systemic injustices, like gender violence and weak primary care infrastructures, that serve to perpetuate poor health outcomes [1]. Furthermore, global legal frameworks, ranging from trade agreements to revised health regulations, can entrench inequalities or be contested and reformed through sustained advocacy. Another point of note is that grassroots mobilization and coalition-building, as seen in cases such as TAC’s work in South Africa, demonstrate that people most affected by injustice can become powerful agents in reshaping health governance. Their advocacy combined litigation, policy engagement, and protest to affirm that health is a matter of human rights and not merely a technical issue that could be bartered or sold as a commodity in a market. Yet the above must be said with caution; some tools and requisites are required for people to be agents in changing health outcomes. The lack of extensive political and civil rights would hamper such efforts, apartheid South Africa being an example itself. Similarly, when looking at Gaza right now, no amount of coalition building for a people suffering injustice would be able to meaningfully change their health governance when the root political determinants of health are too powerful forces. We must be careful and thus clear that while we affirm the role of people to be actors and agents in their destiny, including that of health, we do not serve to minimize the political constraints they are operating under that may override any attempts to combat injustices.

When reflecting on today’s global challenges, it is possible to be struck by how the incomplete realization of health rights calls upon us to confront the enduring colonial legacies present in many systems and neoliberal economic structures that have developed. The successes and failures of the MDG era also show that future progress cannot be assessed through changes in a few metrics alone. We must heed how local capacities, political will, and participatory governance can transform these global frameworks into tangible health equity on the ground.

Socioeconomic inequality’s harms extend to public health and democracy, as it widens existing gaps in how resources, power, and opportunities are distributed, ultimately undermining the notion that all people deserve equal dignity. Steep inequalities contribute to a literal public space in which wealthy elites opt out of public systems, whether education or healthcare. At the same time, marginalized populations are left to rely on the under-resourced institutions [1]. This dynamic intensifies health disparities in tandem with eroding democracy by weakening social cohesion. In Pakistan, for example, some of the negative side effects of this at a whole population level are evident, where large swaths of the upper-middle class rely exclusively on private schools and private healthcare, which draws talent from educators and healthcare workers alike due to more competitive pay. This has rendered the public hospital and schooling systems woefully inadequate, and the difference in content and scale of the service provided in each of their respective spheres can be so vast that there may essentially be two parallel populations in one society. Each interacts with systems so starkly different that they may be wholly unrecognizable to the other. When a minority, be it religious, ethnic, socioeconomic, or other demographic, has the means to shield itself from the consequences of inadequate public services, it is at times less likely to invest in improving those services, thereby perpetuating the cycle of inequality.

During the COVID-19 pandemic, preexisting health inequities in the United States came into the spotlight due to how they manifested. Essential workers, often from lower-income, racially marginalized groups, faced higher exposure risks because remote work was not an option. Many lacked adequate sick leave or health insurance, thus delaying their care options. These patterns are neither accidental nor purely biological but are linked to embedded socioeconomic and racial hierarchies. When adopting a whole-person lens, we can see that race, gender, class, and other axes of identity compound health risks. For example, women racialized as Black individuals in the United States have been previously noted to experience higher rates of maternal mortality than their counterparts racialized as White individuals, hypothesized by some to be not only due to income level but also as a consequence of systemic racial bias in medical settings. Similarly, Palestinian citizens of Israel face numerous barriers to care, including directly prejudicial laws, that result in poorer health outcomes. Brazil also showcased examples of how gender, race, and poverty can all converge together to create barriers that lead to inadequate and delayed care and may ultimately violate the right to life [1].

Intersectionality can thus be seen as a critically important tool in any rights-based framework because it helps uncover how people who inhabit multiple marginalized statuses face more significant hurdles in claiming the rights one is entitled to. Many people’s deaths are not merely the result of a failing hospital but rather a structural outcome rooted in systemic racism, sexism, and poverty. By naming and examining these intersecting factors, advocates in such cases may be able to press for accountability measures that acknowledge both the immediate failures in a patient’s treatment and the more significant institutional drivers of harm [1].

A significant lesson from these last few decades is that a human-rights-based approach to health financing must account for real-world structural inequalities. Health systems cannot be treated merely as marketplaces. Instead, they must be perceived as the social institutions they are and reflect a society’s commitment to equity and dignity that they show to be [1]. From a rights-based standpoint, prioritizing public investment, progressive taxation, and robust regulatory oversight are important tools in ensuring that no individual is denied care due to an inability to pay. Without sufficient government funding, the public health system becomes a safety net only for those who cannot afford private care, reinforcing a two-tiered system. This creates an underfunded public sector that struggles to provide even the most basic services, exacerbating inequalities and undermining democracy.

The issue of trust in public health and science was a pivotal point during COVID-19 [1]. Polarized environments fueled by misinformation acted to weaken public confidence severely. The proliferation of fake news through social media platforms and news outlets with targeted specific audiences helped shape health outcomes in some areas. When people do not trust that health authorities, elected officials, or scientific institutions are working in their best interests, they may reject lifesaving guidance. Building that trust requires transparent, participatory decision-making and the acknowledgment of past betrayals, especially for marginalized communities who have endured discrimination in clinical settings. As the pandemic revealed, trust and equity are intertwined assets, as only when communities feel respected and prioritized by public health systems will they engage fully, whether by seeking care sooner or choosing vaccination to protect themselves and others.
